# Tolerance of three *Aedes albopictus* strains (Diptera: Culicidae) from different geographical origins towards winter temperatures under field conditions in northern Germany

**DOI:** 10.1371/journal.pone.0219553

**Published:** 2019-07-16

**Authors:** Lisa Tippelt, Doreen Werner, Helge Kampen

**Affiliations:** 1 Friedrich-Loeffler-Institut, Federal Research Institute for Animal Health, Greifswald–Insel Riems, Germany; 2 Leibniz Centre for Agricultural Landscape Research, Muencheberg, Germany; University of Cincinnati, UNITED STATES

## Abstract

The continuing spread of the Asian tiger mosquito *Aedes albopictus*, a vector of many arboviruses and some dirofilarial worms, in Europe calls for advanced investigations on its ecological ability to establish and overwinter in temperate, more northern geographic regions. To meet this purpose, eggs of *Ae*. *albopictus* laboratory strains of tropical, subtropical and temperate origin were exposed to field conditions during one or two winter seasons in northeastern Germany. After 1 to 16 weeks of outdoor exposure, eggs were flooded in the laboratory, and the hatching rates were determined. During the winter season 2015/2016, when temperatures reached –10°C, the subtropical strain showed hatching after all time periods while the tropical strain displayed hatching only until two weeks of cold exposure. In the winter season 2016/2017, with temperatures as low as ‒6°C, all three strains produced hatching larvae after all time periods. Both the hatching rates and the hatching behaviour differed between the strains. Larvae of the subtropical and temperate strains hatched in installments over a period of four weeks while the larvae of the tropical strain hatched within a short time period, often one week. The results of the study demonstrate that *Ae*. *albopictus* strains of different, even tropical, origin might be able to survive a central European winter, although this is likely to depend on the specific course of the temperatures. Further studies with different temperature regimes and different mosquito strains are needed to specify these findings.

## Introduction

*Aedes albopictus* (Skuse, 1894), commonly known as the Asian tiger mosquito, is a relatively thermophilic mosquito species with an Asian-Pacific origin [1]. It is not only restricted to tropical areas but also occurs in more temperate regions of eastern Asia [2]. Within a few decades, this species expanded its distribution area to North and South America [3, 4], Africa [5] and Europe [6]. In Europe, it was found for the first time in Albania in 1979 [7], but only spread over the Mediterranean after additional introductions to Italy a decade later [8]. It is now established in at least 19 European countries [6]. The international trade with used tyres and lucky bamboo has played a major role in its global displacement [7, 9, 10].

Due to its aggressive biting behaviour during the day, *Ae*. *albopictus* is a severe nuisance [11]. It uses a broad range of vertebrate hosts as blood sources but preferentially feeds on humans [12, 13]. As it is a competent vector of dirofilarial worms and more than 20 arboviruses [14, 15], it has a high medical and veterinary importance. Recent cases and outbreaks of dengue and chikungunya in Italy, Croatia and France mediated by *Ae*. *albopictus* [16–21] demonstrate that the potential of this species of spreading diseases in Europe is extraordinary.

In Germany, *Ae*. *albopictus* was first found in 2007 in the form of its eggs while monitoring the southwestern federal state of Baden-Wurttemberg [22]. Later on, adult specimens were regularly trapped in the same region [23–25]. All places where specimens were initially found were closely located to motorways, suggesting an importation of adults from southern Europe by vehicles to Germany [25].

In 2014, larvae and pupae of *Ae*. *albopictus* were found for the first time over an extended period of almost three months at a southern German locality [26]. Meanwhile, there is evidence for overwintering [27–29].

*Aedes albopictus* is known to have a high ecological plasticity. Individuals of different geographic origin can react very differently to ecological parameters, including temperature [15]. In tropical areas, for example, the species shows continuous reproduction [30] while it overwinters by diapausing in the egg stage in more temperate areas [31]. Diapausing is triggered by a decrease in temperature and day length [32]. Thus, under certain conditions, females start producing dormant eggs that are better adapted to cold temperatures than non-diapausing eggs [33]. Eggs of individuals from tropical regions are usually not able to diapause as demonstrated for populations from South America [34]. The ability of overwintering is therefore an important trait for establishment of *Ae*. *albopictus* in central Europe.

The present study aims to add further experimental evidence to elucidate the cold tolerance of *Ae*. *albopictus* and its ability to overwinter in central Europe under field conditions. The primary purpose of the study was not to generate high hatching rates as necessary for efficient laboratory rearing but to demonstrate whether hatching after exposure to wintry conditions is possible or not. Even low hatching rates after the winter must be supposed to produce sufficient individuals to build up a population during the next vegetative season and to guarantee local maintenance through the years.

## Material and methods

### Ethics statement

According to EU regulations and national law, no permissions were necessary for conducting the experiments. No endangered or protected species were involved in this study.

### Mosquito rearing

The study was conducted with three laboratory strains of *Ae*. *albopictus*, one from Mauritius, hereafter referred to as ‘tropical strain’, one from Rimini, Italy, hereafter referred to as ‘subtropical strain’, and one from Freiburg, Germany, hereafter referred to as ‘temperate strain’. The tropical and subtropical strains had been kept in the laboratory for numerous generations (about two years) at 25 ± 1°C, 70 ± 5% relative humidity and a light:darkness regime of 12:12 hours. The temperate strain was reared from larval collections made in summer 2015 in Freiburg, southern Germany, and has been held in an insectary at the same conditions for more than a year. For the experiments described, generation F8 was used.

To produce eggs, the mosquitoes were fed on bovine EDTA blood via a membrane feeding system (Hemotek, UK). Wooden spatulas, with one end submerged in stale tap water, or moist filter paper were offered as oviposition supports. All experiments were started with non-diapausing and not cold-acclimated eggs.

### Experimental setup

Spatulas loaded with *Ae*. *albopictus* eggs were transferred into plastic boxes. The boxes were closed but holes were drilled in their lids and sides for ventilation. Several of these boxes were put in a larger plastic container covered with mesh which was positioned on the institute’s premises on the Isle of Riems in the Baltic Sea (Greifswald, Germany, 54.182268, 13.369745) under a bush where it was sheltered from strong winds. A data logger (HOBO Pro v2 temp/RH, Onset Computer Corporation, USA), programmed to measure the temperature and relative humidity every two hours, was also placed in the secondary container. Further spatulas with eggs, stored in plastic beakers in the insectary of the institute at above laboratory rearing conditions, were used as controls.

The eggs were to remain on the spatulas, both outdoors and indoors, for various time periods during wintertime after which they were flooded in the laboratory and checked for larval hatching. Only eggs not older than two weeks and produced in the same blood feeding cycle were used for the study.

#### Winter 2015/2016

In the winter 2015/2016, only the tropical and the subtropical strains were tested. As the tropical strain was newly introduced to the institute and needed some time for producing sufficient numbers of eggs, the study was started at different time points for each of the strains: eggs of the subtropical strain (laid 4–8 December) were brought outdoors 16 December 2015, while the tropical strain eggs (produced 8–11 January) followed 13 January 2016.

For statistical reasons, at least 30 eggs were meant to be processed per spatula in the winter 2015/2016. This minimum number of eggs would allow the detection of a minimum hatching rate of at least 10% with a 95% confidence interval under the assumption of a bimodal distribution. The number of eggs actually used ranged from a minimum of 34 (tropical strain) to a maximum of 1,340 eggs per spatula (subtropical strain) (Table 1).

**Table 1:** Number of eggs used in the winter study 2015/2016.

Mosquito strain	Experimental group	Mean number of eggs per spatula (min.–max.)
tropical strain	controls	77 (50–144)
	treatments	57 (34–110)
subtropical strain	controls	661 (146–1,340)
	treatments	730 (376–1,191)

After 1, 2, 4, 8, 12, 14 and 16 weeks of being outside, the spatulas were brought into the laboratory and flooded for larval hatching. Before flooding, eggs were counted. If the spatulas were covered with high numbers of eggs, pictures were taken and eggs were counted with the software NIS Elements (Nikon, Japan) by clicking on the picture. Empty egg shells, possibly caused by hatching in reaction to intruding precipitation water during the experiment, were excluded from the total score of eggs. Flooding was done by submerging the spatulas in containers with stale tap water. Hatched larvae were also counted and removed from the flooding containers during the process of counting. Larvae found dead after successful hatching were evaluated like living ones.

Flooding of samples was performed in two steps. First, eggs were flooded for four weeks in the same water. After that, the spatulas were removed from the water and dried for one week in the laboratory. The flooding water was filtrated through common laboratory filter paper for retaining non-hatched eggs potentially detached and floating in the water. With a brush, these eggs were re-attached onto the spatulas. Second, all eggs hitherto non-hatched were flooded again in stale tap water for another two weeks after the drying period. Unhatched eggs were not checked for being viable or not.

#### Winter 2016/2017

In the winter season 2016/2017, all three *Ae*. *albopictus* strains were used. The methodology was slightly changed: instead of wooden spatulas, the blood-fed mosquito females were offered moist filter paper for oviposition as this substrate turned out to be favoured and, thus, to be more effective in producing high numbers of eggs. Furthermore, the number of eggs used in the experiment was standardised: some 30 of the produced eggs were transferred from the filter paper onto a wooden spatula by means of a paint brush. Per time period, two of these spatulas from the same oviposition event were tested under exactly the same conditions, resulting in one replication per time period and temperature. Thus, the number of eggs per treatments or controls tested at the same time was increased to about 60 as compared to the winter 2015/2016 study, allowing the detection of a minimum hatching rate of 5% with a 95% confidence interval under the assumption of a bimodal distribution.

Furthermore, the experiments started at the same time (7 December 2016) for all three mosquito strains involved. Eggs used had been laid between 20 November and 1 December 2016. Time periods tested and the flooding regime were also changed: eggs were flooded after 2, 5, 8, 12 and 16 weeks for two weeks, dried for one week and flooded for another week. For both floodings, stale tap water was used as hatching medium.

### Statistical analysis

For evaluating the hatching success, relative proportions of hatched larvae were calculated per study period and strain. A factorial design was chosen for the experiments as three different factors would influence the response variable, the hatching rate. The first factor is the ‘strain’ with two different levels (tropical, subtropical) in the winter 2015/2016 and three levels (tropical, subtropical, temperate) in the winter 2016/2017. The second factor (‘exposure’) is the time period the eggs were exposed to low temperatures. The first winter comprised six and the second winter five time period levels. The last factor is the ‘group’ and defines the assignment of eggs to the levels ‘treatment’ and ‘control’.

For the winter 2015/2016, we decided to only compare hatching results within a strain as the experiments with the two strains could not be started contemporaneously and no replicates were run. Hence, hatching results of all exposure times were summarised and compared using a Kruskal-Wallis test. In the winter 2016/2017, a direct comparison between the strains was possible. Therefore, all factors and their impacts on the hatching rate could be tested applying a multifactorial ANOVA. For meeting the assumptions of the ANOVA, data were square root-transformed. Subsequently, a Tukey’s honestly significant different (HSD) post hoc test was applied for revealing the significant levels within the various factors. All statistical tests were made with the software R (version 3.4.1.) without using additional packages.

## Results

**Fig 1:**
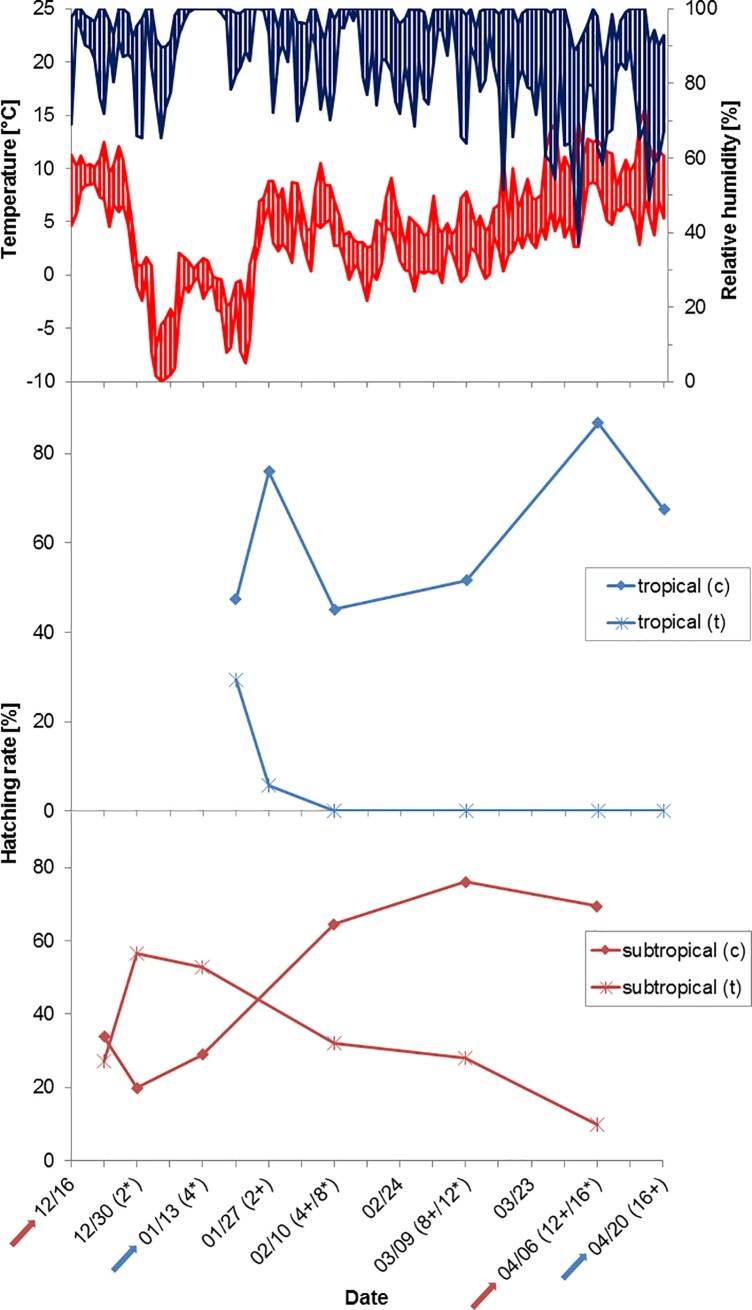
Temperature (red) and humidity profiles (blue) at the study site on the Isle of Riems in the winter 2015/2016 (top) with hatching rates of the tropical (middle, blue lines) and subtropical strain (bottom, red lines); c = controls, t = treatments, no replicates run. Arrows mark the start/end of the experiments with the two mosquito strains. The numbers in parentheses, representing dates of withdrawal of a subsample for flooding, indicate the number of weeks elapsed since the start of the experiment (* = subtropical strain, + = tropical strain).

The 2015/2016 experiment started at temperatures of 11°C (16 December 2015, subtropical strain) and –2°C (13 January 2016, tropical strain), respectively, as measured by the data logger in the secondary container. Two cold periods occurred in early and late January, respectively, with the first lying before the outdoor exposure of the tropical strain: this first cold period lasted from 2 to 7 January with temperatures of almost –10°C for 2 hours, each, at two days and a temperature maximum of –3°C (Fig 1A, S1 Table). The second cold period lasted from 18 to 23 January with a temperature decrease to –8°C for 4 hours and a maximum temperature of –3°C. At the end of the experiment, April 6 (subtropical strain) and April 20 (tropical strain), temperatures measured 13°C and 22°C, respectively. Relative humidity fluctuated between 37% (minimum) and 100% (maximum), with higher variation from early March onwards, but mostly remaining between 70 and 100% (Fig 1A).

**Fig 2:**
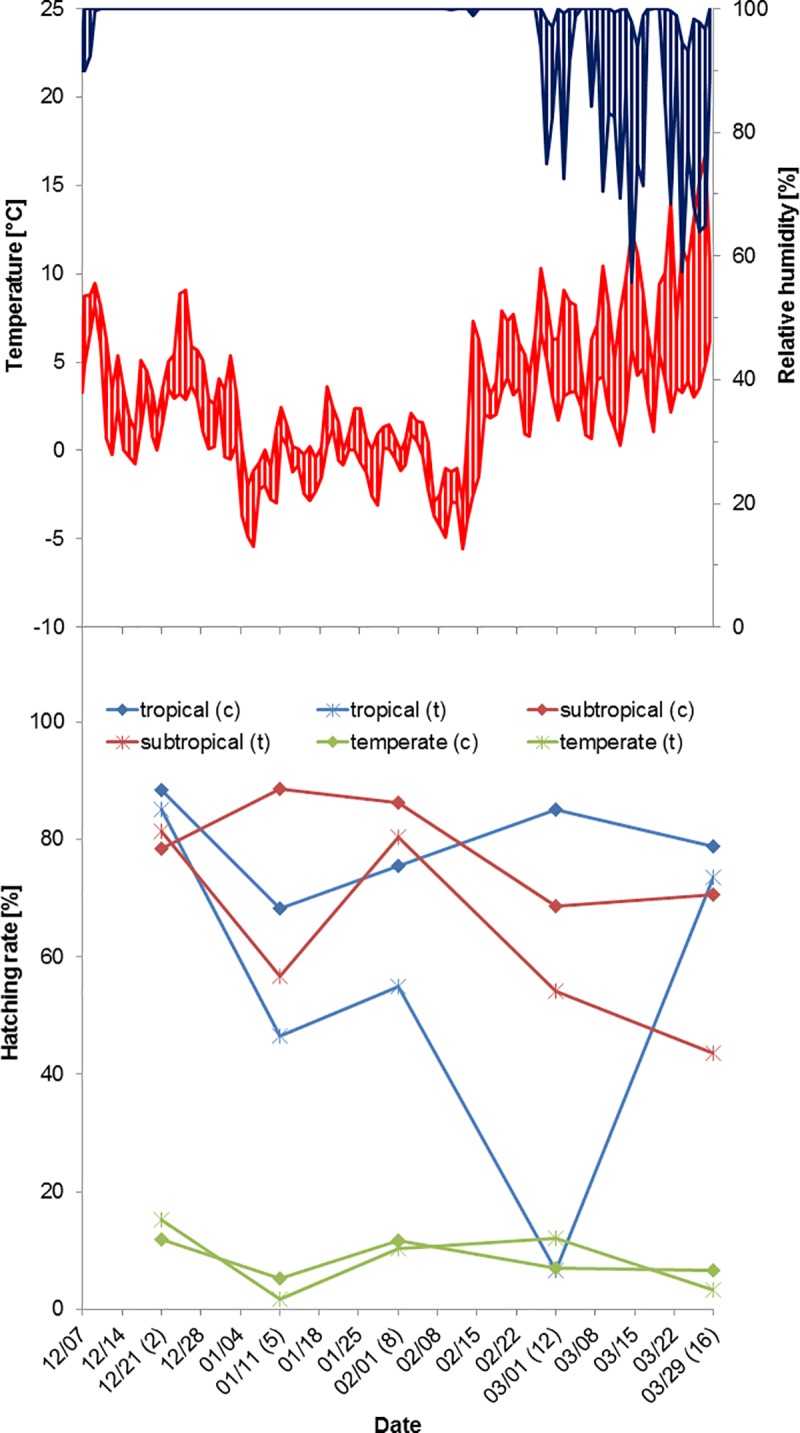
Temperature (red) and humidity profiles (blue) at the study site on the Isle of Riems in the winter 2016/2017 (top) with mean hatching rates of all strains (c = controls, t = treatments); one replicate run. The numbers in parentheses, representing dates of withdrawal of subsamples for flooding, indicate the number of weeks elapsed since the start of the experiment.

The temperature at the beginning of the winter 2016/2017 study was 3°C. It decreased to a minimum of almost –6°C and again showed two colder periods, one in early January and one by mid-February (Fig 2A, S1 Table). The first cold period lasted from 5 to 7 January and was characterised by temperatures as low as –5°C for 4 hours, whereas the second cold spell lasted from 8 to 14 February with a minimum temperature of almost –6°C for 4 hours and a maximum temperature of –1°C. At the end of the experiment (29 March), the temperature was measured as 10°C. Relative humidity varied between 56% (minimum) and 100% (maximum), with values close to 100% during the whole winter season and greater fluctuations starting only in early March (Fig 2A).

Regarding the tropical strain, larvae hatched from eggs kept outside only after one and two weeks of exposure in the winter 2015/2016 (Fig 1B). Interestingly, the two weeks´ exposure sample produced larvae only after the drying period, but not during the four weeks’ flooding period. Hatching rates of controls were usually around 50% and almost 90% in one case. In the winter 2016/2017, this strain showed hatching in all treatments, although with very low hatching rates after 12 weeks of cold exposure (Fig 2B). Hatching results of controls fluctuated between 70% and nearly 90% (S3 Table).

The cohort treatment eggs of the subtropical strain showed hatching after all time periods in both winters. However, the longer the eggs had been in the field, the lower the hatching rates got (Fig 1C). After 16 weeks outside in the winter 2015/2016, larvae hatched from only 10% of the eggs. Such a pattern was not observable in the winter 2016/2017 when hatching rates of treatments did not decrease with time of cold temperature exposure (Fig 2B). Hatching rates in the controls peaked at ca. 76% in the winter 2015/2016 and almost 90% in the winter 2016/2017 (Fig 1C, S2 and S3 Tables).

The *Ae*. *albopictus* strain from Freiburg, Germany, which was included in the study only in the winter 2016/2017, showed larval hatching in all treatment groups, although rates were much lower than in the other two strains (Fig 2B). However, also the controls presented with much lower hatching rates than the controls of the other strains (Fig 2B).

The statistical comparison of treatments and controls of the winter 2015/2016 showed significantly different values for the tropical strain (χ^2^ = 8.61, p < 0.01), but not for the subtropical strain (χ^2^ = 1.64, p > 0.05). The results of the ANOVA suggests that all studied factors (‘strain’, ‘exposure’ and ‘group’) as well as their interactions had a significant influence on the hatching rate (Table 2).

**Table 2:** Result of the ANOVA relating hatching results to the factors ‘strain’, ‘exposure’ and ‘group’.

Factor	F-value	p-value
strain	358.57	< 0.001*
group	31.99	< 0.001*
exposure	10.98	< 0.001*
strain : group	6.15	< 0.01*
strain : exposure	5.35	< 0.001*
exposure : group	4.57	< 0.01*
strain : exposure : group	5.88	< 0.001*

* significant

The Tukey’s HSD post hoc test indicates that the temperate strain is responsible for the significant result within the factor ‘strain’ (p < 0.001), while the pairwise comparison between the exposure time of 2 and 12 weeks produces the significance within the factor ‘exposure’ (p < 0.001). If the pairwise comparisons of the treatments and controls of the same strain are considered, it can be shown that the low hatching result of the treatments of the tropical strain exposed for 12 weeks outside produced the significant result. Pairwise comparisons between the controls of different exposure times were insignificant for all strains.

**Fig 3:**
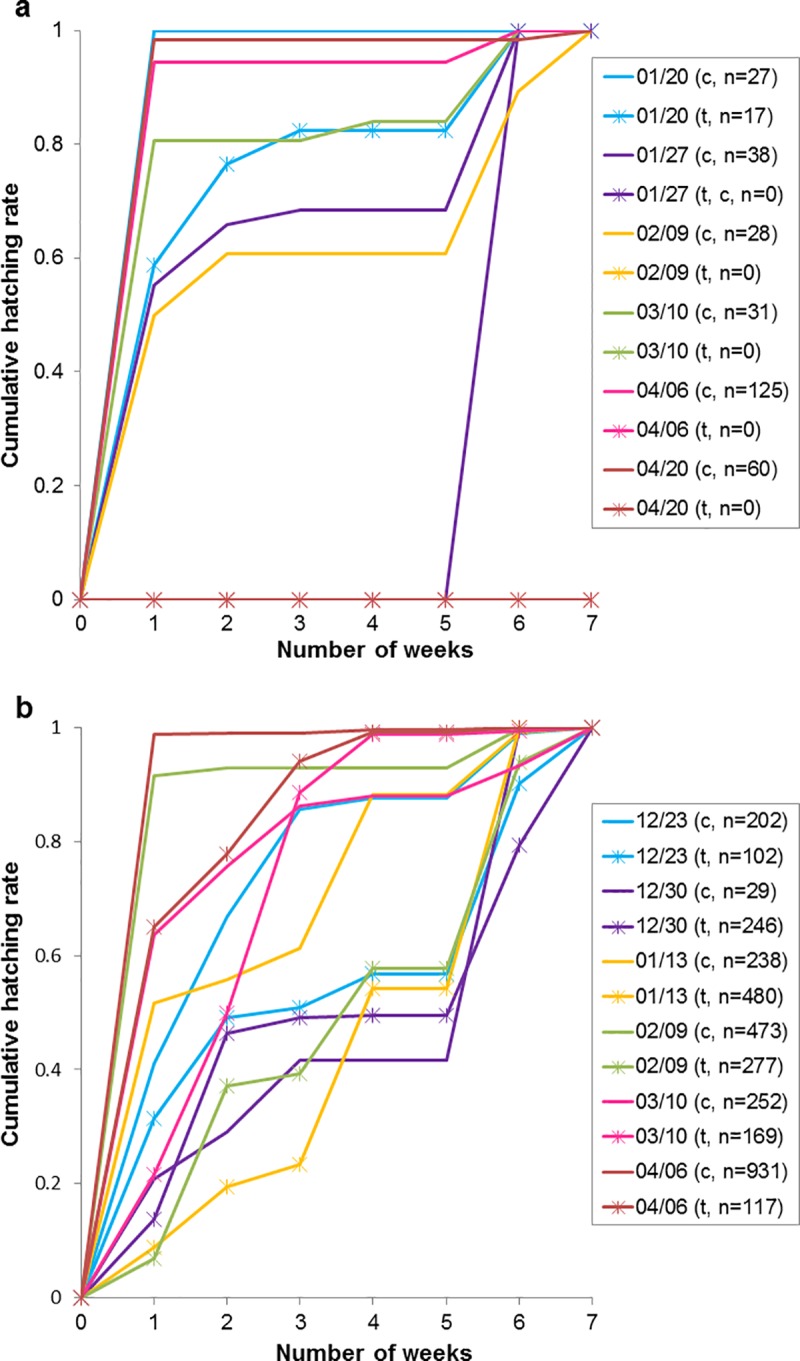
Cumulative hatching rates of the tropical strain (a) and of the subtropical strain (b) in the winter 2015/2016 (c = controls, t = treatments). The numbers in parentheses indicate the total number of hatched larvae. The cumulative hatching rates add up to the value ‘1’ which represents the final hatching rates as presented in Fig 1B and 1C, respectively.

In addition to the hatching rates, the hatching pattern differed between the two *Ae*. *albopictus* strains used in the winter season 2015/2016. Larvae of the tropical strain often hatched within a short time window after flooding (Fig 3A, S4 Table). Consequently, hatching events often only occurred at the beginning of the four weeks’ and the two weeks´ flooding periods. The only exception was registered at the end of the two weeks´ flooding period of the last treatment when one late larva hatched. By contrast, larvae of the subtropical strain hatched in installments, i.e. hatching occurred over an extended time period. Most samples of this strain showed a hatching peak at the beginning and after three weeks of the four weeks´ flooding period (Fig 3B, S4 Table). Furthermore, eggs of both strains showed a higher hatching response after the drying period than during the four weeks´ flooding period.

Increased hatching at the beginning of the flooding periods was also noticed in the winter season 2016/2017 (S5 Table). However, a clear difference in hatching behaviour between the tropical and the subtropical strains, as observed in the winter season 2015/2016, was hardly noticeable, probably due to the shorter initial flooding period.

## Discussion

The results of the study support the assumption that the cold hardiness of *Ae*. *albopictus* eggs is dependent on the geographic origin of the strain. Specifically, *Ae*. *albopictus* strains originating from a warmer climate have a lower tolerance against low temperatures than those from regions with a cooler climate [33, 35, 36].

Not surprisingly, the tropical strain used in this study had a worse performance in the winter 2015/2016 than the subtropical strain. However, hatching of the tropical strain occurred in all treatments in the winter 2016/2017, although temperatures had temporarily gone down to –6°C and were only slightly above those of the winter 2015/2016. These completely different hatching results cannot only be linked to the innate temperature tolerance of the strain but seems rather related to the phenomenon of cold acclimation. Cold tolerance can artificially and naturally be achieved by exposing eggs to low temperatures above freezing point and has a major effect on hatching rates, as demonstrated by Hanson & Craig [37]. These authors also showed that the eggs of temperate *Ae*. *albopictus* that had been acclimated for a long time had higher hatching rates than those acclimated to the same temperature for a short time. This did not account for eggs of the used tropical strain. However, the apparent absence of cold-acclimation in the tropical strain could also have been caused by exposing the eggs to a temperature of –10°C which is lethal to non-acclimated eggs of tropical *Ae*. *albopictus* even after short exposure [36]. Cold acclimation could also not be shown after exposure to –2°C [38]. As stressed in the methodological description of the experiments, eggs had not artificially been acclimated to low temperatures prior to the study as done in other experiments [37, 39]. The available time period in which eggs could natural cold acclimation greatly differed between the winters 2015/2016 and 2016/2017. In the winter 2015/2016, eggs of the tropical strain were transferred from laboratory conditions to an outside temperature of –2°C in mid-January. Therefore, practically no cold acclimation was possible. By contrast, temperatures were unusually high in December 2015, when eggs of the subtropical strain were brought outside, and started dropping below 5°C not before the end of December. These divergent conditions might have led to the huge differences in hatching rates of the tropical and subtropical strains.

As opposed to the winter 2015/2016, the time period available for cold acclimation was considerably longer for the tropical strain in the winter 2016/2017 when the temperature measured 3°C at the beginning of the experiment and went down to subzero values not before early January, about one month after the start of the experiment. Consequently, hatching rates were much higher and more similar to the subtropical strain than in the winter before. To our knowledge, this is the first description of cold acclimation in a tropical strain of *Ae*. *albopictus*.

In addition, the measured minimum temperature had an influence on the hatching rates of the tropical strain, as shown by the results of the statistical tests in both winters. Naturally, the tropical strain is not exposed to low temperatures, so no selection pressure exists in favour of the development of cold-resistant eggs. Thomas et al. [36], who used the same *Ae*. *albopictus* tropical and subtropical strains experimentally, had previously shown that the tropical strain produced hatching results more similar to another tropical mosquito species, *Ae*. *aegypti* (Linnaeus, 1762), than to the subtropical *Ae*. *albopictus* strain. They depicted that hatching of the tropical strain did not occur anymore at –7°C and –10°C, when eggs were exposed to these temperatures for longer than one hour.

In the winter 2015/2016, the tropical strain was exposed to a minimum temperature of –8°C for four hours. Nevertheless, two larvae succeeded in hatching from 35 eggs. Thomas et al. [36] described such a surprising hatching event in the same mosquito strain, with one hatched larva out of 20 eggs after exposure to –10°C for one hour, and Hanson [40] reported hatching rates lower than 14% for a tropical strain from Malaysia exposed to a minimum temperature of –10°C for an unknown duration during a field experiment in Japan. In the present study, the minimum temperature of –6°C, measured for less than two hours in the winter 2016/2017, also caused a considerable decrease in the hatching rate of the tropical strain in the 12 weeks´ treatments followed by a certain recovery. This conspicuous hatching rate was in stark contrast to all other hatching rates of the strain, causing a huge variance with significant divergences within this strain. This is contrary to findings of other studies showing only insignificant variation of the hatching rates, followed by cessation of hatching [36, 38, 40, 41]. Therefore, a random effect caused by the overall small sample size cannot be excluded, although the effect occurred in both parallel treatments. Further studies could show if this effect can be replicated on a large scale or had only randomly been produced.

Despite the exceptional low hatching rate of the 12 weeks´ treatment, the present study clearly shows that the lethal temperature or the lethal exposure time was not reached for the eggs of the tropical strain in the winter 2016/2017, in contrast to the winter 2015/2016. In summary, it is very likely that the interaction of temperature minimum and duration, or a putative cold acclimation, had contributed to the hatching results of the tropical strain in the present study.

With high hatching rates, the subtropical strain presented surprisingly robust in a realistic central European winter scenario with fluctuating temperatures and humidity, although temperatures went down to –10°C in the winter 2015/2016. Laboratory experiments have shown that eggs of the same laboratory strain could endure temperatures of –10°C for at least 24 hours and still exhibited hatching after exposure to temperatures as low as –12°C for four hours [36]. Interestingly, only the treatments in the winter 2016/2017 were significantly different from their controls while the differences between treatments and controls were not significant in the winter 2015/2016 (Table 2). Yet, this is no evidence for different temperature tolerances in the two winter seasons but might be generated by the different hatching rates of the controls in the winter 2015/2016: Controls flooded after the first three time intervals in the winter 2015/2016 had much lower hatching rates as compared to the high hatching rates of the controls flooded later (Fig 1C). Such restriction caused by an imbalance of hatching rates did not occur in the winter 2016/2017. The data lead to the conclusion that below survival temperatures were not reached for the subtropical strain at the site of exposure in any of the two winters.

The temperate strain showed a poor hatching performance throughout the experiments. Neither the treatments nor the controls reached hatching rates higher than 18%. A possible explanation for this observation could be the incomplete adaptation of this strain to laboratory conditions as it had only recently been acquired from the field, with only few generations passed in the laboratory environment before the start of the experiments. This phenomenon of low winter hatching rates in newly established laboratory strains is also known from other laboratories. Due to this unusual hatching behaviour, the explanatory power of the comparisons of treatments and controls both within this strain and with other strains is limited. However, despite the low hatching rates, the temperate strain showed hatching after all treatments, suggesting a certain survival potential of the population under low temperatures. Interestingly, the statistical analysis of the hatching results of the temperate strain only leads to insignificant influences of exposure time and assignment to control or treatment group. This could be interpreted as a consequence of the small differences between the hatching rates of treatments and controls but also as a limitation of the study due to the small overall sample sizes. The treatments of the temperate strain showed no significant cold-related decrease in the hatching rate irrespective of exposure time to low temperatures. Notwithstanding, a higher tolerance of the temperate strain towards cold temperatures is just speculative and possibly obscured by the overall low hatching rates of this strain.

Due to the local experimental conditions, the present study has two major limitations with respect to generalisation. First, the temperature tolerance of the strains was tested at one particular location only. Hawley et al. [39], for example, could show that the hatching results differed at different localities in one and the same state of the USA, and that the location with the lowest temperature minimum did not necessarily produce the lowest hatching rates. A comparison with regions with harsher winter conditions would therefore be interesting to conduct in the future. In the present study, eggs were kept in boxes at a location protected from wind but not from temperature. These conditions of egg storage may be comparable to the overwintering of eggs attached to the inner walls of tyres. It could be shown that temperatures only slightly differed inside and outside the tyres [33]. However, *Ae*. *albopictus* eggs can be found in a huge variety of natural and artificial containers [35, 42], the latter including underground constructions such as sewage systems and cesspits [43, 44]. These must be considered to provide significantly more shelter from adverse winter conditions than our experimental setup, which gives reason to assume that eggs surviving our scenario would have also survived in sheltered places under natural conditions.

Second, under natural conditions, eggs of *Ae*. *albopictus* from non-tropical regions endure low temperatures through diapausing eggs. In this study, low temperature tolerance was only tested for non-diapausing eggs. It can be expected that hatching results of diapausing eggs would be even higher, as indicated for field conditions by Hanson & Craig [37] and for laboratory conditions by Thomas et al. [36]. In summary, the second point rather emphasises the relevance of our results: even non-diapausing eggs of certain strains of *Ae*. *albopictus* are able to survive low temperature periods.

Our experiments suggest that age had no impact on the hatching results as no significant decrease became evident in all controls of all three strains. However, depending on the hatching conditions, egg age might affect hatching rates. Mogi [41] could show for *Ae*. *albopictus* strains from different eastern Asian and Pacific islands that hatching rates of some strains flooded in water supplemented by bacterial broth were as high as 80 to 90% after 60 to 90 days after oviposition but dropped to 20 to 30% after 120 days after oviposition. Zheng et al. [45] who flooded eggs in bacterial broth found that eggs of three months’ age had a higher average hatching rate than eggs flooded after one or two months. Later than three months, the hatching rates gradually decreased until zero in week 24 which was much later than observed by Mogi [41]. In contrast to that, Gubler [46] determined 243 days as the maximum survival time of *Ae*. *albopictus* eggs continuously flooded with tap water. In the present study, the high hatching rates observed in the controls of the tropical and subtropical strains flooded after four months in both winters suggest that this dropping point was not yet reached, and eggs remained viable until the end of the experiment.

The first overwintering of *Ae*. *albopictus* in Germany is assumed to have occurred from 2014 to 2015, based on the finding of *Ae*. *albopictus* developmental stages in Freiburg, federal state of Baden-Wuerttemberg, early in the season 2015 at the very same location as they had occurred the year before [44]. Recently, Pluskota et al. [27] demonstrated overwintering of *Ae*. *albopictus* in Germany by showing that eggs collected in summer were able to produce larvae after winter field exposure in the Black Forest Mountains in southwestern Germany. The authors missed presenting data on the hatching rate, making a comparison with our data impossible. Notably, the winter season 2015/2016 in Germany and particularly in its southwestern part was characterised by extremely mild temperatures and only short periods below freezing point [47]. Instead of winter temperatures at the very location of egg exposure, Pluskota et al. [26] presented mean temperatures of January measured by a meteorological station about 37 km away. However, when considering survival or extinction, the microclimate, i.e. the temperature and humidity at the exact position of egg location, is decisive. Furthermore, minimum temperatures are possibly even more important for assessing cold tolerance than mean temperatures, as also stressed by Thomas et al. [36]. Single events of extreme temperatures could have a crucial impact on overwintering because they can exceed physiological limits of the species and therefore cause irreversible damage to the eggs.

*Aedes albopictus* appears to be able to adapt to a changing environment very quickly [15]. Its drought-resistant eggs have facilitated its displacement over long distances, and its preference for artificial containers have enabled conquering urban and suburban areas providing optimal feeding and developmental conditions. Globalisation as well as climate change favour the spread of this species [15], although still in 2007, Benedict et al. [48] considered the risk low. By contrast, more recent models suggest a high risk of establishment of this species in western and southern parts of Germany [49–51]. One of them even predicts almost the whole of Germany as suitable for *Ae*. *albopictus* [52]. However, the binary presentation of the predictions (differentiated into unsuitable and suitable areas only) do not allow any statement about the gradual suitability of areas and their various probabilities of being colonized after introduction.

## Conclusions

Although, as opposed to the winter study 2016/2017, the winter study 2015/2016 was not standardised and therefore not statistically analysable, both studies provide evidence of *Ae*. *albopictus* egg survival under natural winter conditions in parts of Germany, although shown for relatively mild winter climates only. With respect to the recorded minimum temperature of –10°C, the hatching results of the winter 2015/16 may, however, allow a transfer of results to wider parts of Germany.

Despite the differences in hatching rates, our results show that all three *Ae*. *albopictus* strains tested are principally able to survive ordinary German winters, i.e. winters without prolonged severe cold periods, albeit the tropical one only survived one complete winter season. *Aedes albopictus* occurrence has not been shown so far at the selected study location, but is conceivable under present climatic conditions as its future spread over the whole of Germany is considered possible given progressing climate warming [52]. Consequently, this invasive vector species must be expected to have survival potential in a much wider area than its current distribution range.

It can be assumed that most of the *Ae*. *albopictus* specimens found in the field in Germany have been introduced from southern Europe or are descendants from those [24–26]. The Italian population used in this study and possibly representative for other southern European strains, performed particularly well in both winters and appears to have a high cold tolerance. Future studies in the laboratory and in the field will show if *Ae*. *albopictus* is able to cope with central European winter temperatures in the long term and to become permanently established in Germany and other more northern European countries. Practically, this will hopefully also be hampered by control actions which have locally been implemented already in Germany [27].

## Supporting information

S1 Table Temperatures and relative humidities recorded during the winters 2015/2016 and 2016/2017. (XLSX)

S2 Table Total numbers of eggs, hatched larvae and hatching rates obtained for the tropical and subtropical strains in the winter 2015/2016. (XLSX)

S3 Table Total numbers of eggs, hatched larvae and hatching rates for the tropical, subtropical and temperate strains obtained in the winter 2016/2017. (XLSX)

S4 Table Cumulative numbers and proportions of hatched larvae per week obtained for the tropical and subtropical strains in the winter 2015/2016 (c = controls, t = treatments). (XLSX)

S5 Table Cumulative numbers and proportions of hatched larvae per week obtained for the tropical, subtropical and temperate strains in the winter 2016/2017 (c = controls, t = treatments). (XLSX)
